# Ferroptosis in periodontitis: mechanisms, impacts, and systemic connections

**DOI:** 10.1038/s41420-025-02550-5

**Published:** 2025-06-20

**Authors:** Peng Guan, Qijun Ruan, Jiatong Li, Mengying Xi, Weijuan Qi, Kang I. Ko, Jia Ni

**Affiliations:** 1 Department of Periodontics, Stomatological Hospital, School of Stomatology, Southern Medical University, Guangzhou, China; 2 Center of Stomatology, The Second Affiliated Hospital, Jiangxi Medical College, Nanchang University, Nanchang, China; 3 JXHC Key Laboratory of Periodontology (The Second Affiliated Hospital of Nanchang University), Nanchang, China; 4 The Institute of Periodontal Disease, Nanchang University, Nanchang, China; 5 Department of Periodontics, School of Dental Medicine, University of Pennsylvania, Philadelphia, PA USA

**Keywords:** Cell death, Oral diseases

## Abstract

Periodontitis is a chronic inflammatory disease initiated by plaque microorganisms, with the regulatory mechanisms of its progression being a primary research focus. Ferroptosis, a unique form of cell death driven by iron-dependent lipid peroxidation, has been increasingly recognized for its crucial role in modulating chronic inflammation. This study focused on the molecular mechanisms by which plaque microorganisms and the inflammatory microenvironment trigger ferroptosis in periodontal cells, elucidating how ferroptosis in these cells promotes periodontitis progression. Additionally, the potential exacerbation of periodontitis through ferroptosis in systemic diseases such as Alzheimer’s disease, nonalcoholic steatohepatitis, chronic obstructive pulmonary disease, and type 2 diabetes is discussed. This review aims to provide new theoretical foundations and strategies for the treatment of periodontitis.

## Facts

- The inflammatory microenvironment in periodontitis promotes ferritin accumulation and lipid peroxidation across various periodontal cell types, ultimately triggering ferroptosis.
- Ferroptosis in periodontal cells amplifies the inflammatory response within tissues, thereby exacerbating periodontitis severity.
- Periodontitis is significantly associated with various systemic diseases, such as Alzheimer’s disease, nonalcoholic fatty liver disease, chronic obstructive pulmonary disease, and diabetes. However, the precise regulatory mechanisms underlying these connections remain largely unexplored.

## Questions

- How does periodontitis induce ferroptosis in various cells within periodontal tissues? What are the key molecular mechanisms involved?
- What impact does ferroptosis in periodontal cells have on periodontal inflammation?
- Does ferroptosis mediate the comorbidity between periodontitis and systemic diseases? What are the potential molecular mechanisms involved?

## Introduction

Periodontitis is a chronic inflammatory disease primarily driven by plaque-associated microbial dysbiosis, leading to the progressive deterioration of periodontal support structures, including the gingiva, periodontal ligament, cementum, and alveolar bone [1, 2]. As a major cause of tooth loss in adults, periodontitis significantly impacts both oral and systemic health [3]; the disease is characterized by persistent inflammation, immune cell infiltration, and the destruction of supporting tissues, ultimately leading to impaired function and compromised oral health [4].

Ferroptosis, an iron-dependent cell death pathway characterized by phospholipid peroxidation and plasma membrane damage [5, 6], has emerged as a key player in various inflammatory diseases [7, 8]. Notably, ferroptosis is implicated in periodontitis progression, although the precise mechanisms remain incompletely understood [9].

Within diseased periodontium, the persistent upregulation of inflammatory cytokines and reactive oxygen species (ROS) and disrupted iron metabolism collectively create conditions favourable for ferroptosis in various cells, including periodontal and immune cells [10, 11]. This pathological environment induces ferroptosis in fibroblasts, periodontal ligament cells, osteocytes, and immune cells such as macrophages, T cells, and neutrophils, contributing to tissue degradation and increased inflammation [12–14]. Additionally, ferroptosis-related mechanisms in periodontitis may also be linked to systemic diseases such as Alzheimer’s disease, nonalcoholic fatty liver disease, chronic obstructive pulmonary disease, and diabetes.

A review of the current literature revealed that in periodontitis, the mechanisms of ferroptosis in different types of periodontal cells (including periodontal immune cells, fibroblasts, osteocytes, and periodontal stem cells) vary [9, 15, 16]. However, there has been no comprehensive summary or discussion of this topic in the literature. This review classifies and discusses the mechanisms of ferroptosis in different cell types, which represents one of the innovations of this article. Furthermore, ferroptosis induced by periodontitis may not be confined to the periodontal tissues themselves. Periodontal pathogens may trigger ferroptosis in the cells of distant organs (such as the liver and lungs) through the systemic circulation, thereby promoting the development of systemic diseases. This review also explores the mechanisms by which periodontitis-induced ferroptosis affects distant organs.

## Mechanisms of ferroptosis

**Fig. 1:**
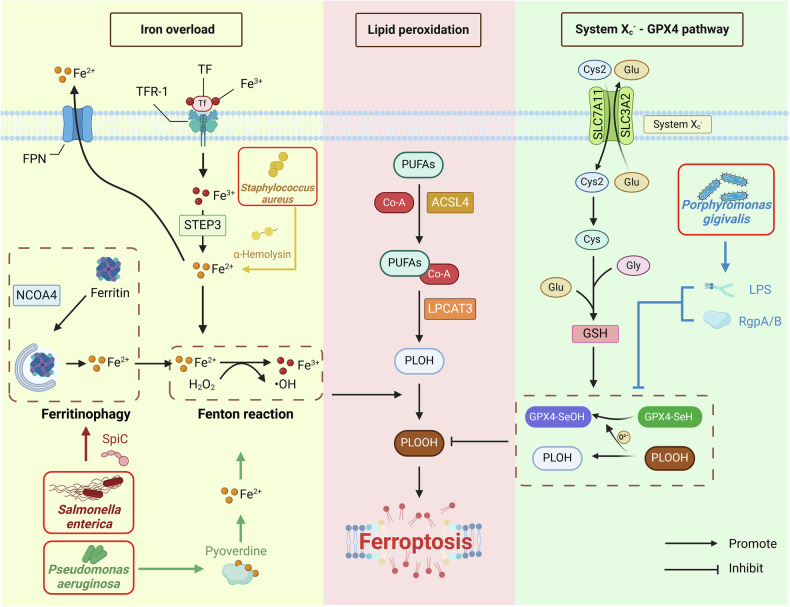
Key mechanisms of ferroptosis. The primary pathways driving ferroptosis include iron overload, lipid peroxidation, and the disruption of the antioxidant system, particularly the Xc⁻–GPX4 pathway. During iron overload, Fe²⁺ oxidizes lipids via the Fenton reaction to form lipid peroxides. Enzymatic pathways involving ACSL4, LPCAT3, LOX, and COX also contribute to lipid peroxidation, leading to ferroptosis. Antioxidant defence mechanisms, such as GPX4 in conjunction with GSH, can reduce oxidized hydroperoxide (PLOOH) levels, preventing ferroptosis.

Ferroptosis is an iron-dependent form of cell death characterized by lipid peroxide accumulation driven by disrupted iron metabolism and oxidative damage [5, 17]. This section will detail the key mechanisms of ferroptosis, including iron overload, lipid peroxidation and the dysregulation of the antioxidant system (particularly the system Xc⁻–GPX4 pathway), highlighting their interactions that culminate in cell death and tissue damage (Fig. 1).

### Iron overload

Intracellular iron overload typically begins with the abnormal accumulation of Fe²⁺ [5, 18]. Under normal conditions, extracellular circulating Fe³⁺ is transported into the cell via the transferrin (TF)/transferrin receptor (TFR-1) system and then reduced to Fe²⁺ by the metal reductase six-transmembrane epithelial antigen of prostate 3 (STEAP3), subsequently entering the intracellular labile iron pool [19]. Intracellular Fe²⁺ can be exported outside the cell via ferroportin (FPN), the only known iron-exporting membrane protein in mammals [20]. The upregulation of transferrin receptor (TFR-1) expression is a key feature in cells undergoing ferroptosis and can be reliably identified by antibody-based detection [21].

Excess intracellular Fe²⁺ reacts with hydrogen peroxide (H₂O₂) via the Fenton reaction, generating highly reactive hydroxyl radicals (•OH), a potent form of ROS that drives ferroptosis. The primary mechanisms leading to Fe²⁺ overload include upregulated ferritinophagy, where nuclear receptor coactivator 4 (NCOA4) binds to ferritin and directs it to lysosomes for degradation, releasing Fe²⁺ [22]. Additionally, increased Fe³⁺ uptake or decreased release can contribute to iron overload, as the abnormal expression of hepcidin, a key regulator of iron metabolism, disrupts iron homoeostasis. Furthermore, the upregulation of the expression of ferroptosis-promoting factors, such as haem oxygenase-1 (HO-1), results in the degradation of haem and the release of more Fe²⁺, further promoting ferroptosis [19, 23].

### Lipid peroxidation

Lipid peroxidation is one of the main pathological mechanisms of ferroptosis and involves the oxidation of polyunsaturated fatty acids (PUFAs) in the cell membrane, leading to membrane lipid damage. This process can be initiated via two pathways: nonenzymatic and enzymatic reactions [22].

The nonenzymatic pathway is mediated by the Fenton reaction, during which iron catalyzes the conversion of hydrogen peroxide (H₂O₂) into highly reactive •OH [24]. •OH initiates a lipid radical chain reaction by abstracting hydrogen atoms from the carbon chains of PUFAs in the plasma membrane, resulting in rapid oxidation and degradation of membrane lipids [5, 25].

The enzymatic lipid peroxidation pathway is primarily mediated by enzymes such as lipoxygenase (LOX) and cyclooxygenase (COX). Long-chain acyl-CoA synthetase 4 (ACSL4) and lysophosphatidylcholine acyltransferase 3 (LPCAT3) play critical roles in this process [22, 26]. ACSL4 promotes the acylation of free PUFAs, linking them with coenzyme A (Co-A), while lysophosphatidylcholine acyltransferase 3 (LPCAT3) incorporates PUFA-CoA into membrane phospholipids, increasing the vulnerability of the plasma membrane [26]. LOX and COX subsequently catalyze the oxidation of polyunsaturated fatty acids (FAs), generating lipid peroxides and resulting in membrane damage. Therefore, the concurrent detection of lipid peroxide products such as 4-hydroxy-2-nonenal (4-HNE) or malondialdehyde (MDA) is essential for determining ferroptotic status [27].

### System Xc^−^–GPX4 pathway

The system Xc^−^–GPX4 pathway is a key intracellular antioxidant and antiferroptosis mechanism [28, 29]. This pathway begins with the amino acid transport system System Xc^−^, which is composed of SLC7A11 and SLC3A2 subunits and mediates the exchange of extracellular cystine for intracellular glutamate [30]. Cystine is then reduced to cysteine, which combines with glutamate and glycine to form glutathione (GSH), a critical intracellular antioxidant that scavenges free radicals and peroxides, thereby protecting cells from oxidative damage [31].

GPX4 is a crucial enzyme for inhibiting ferroptosis, as it blocks the cascade of lipid peroxidation; its antioxidant activity depends on selenium, which is present in the form of selenocysteine [32]. The low electronegativity of selenium allows it to efficiently reduce oxidized phospholipid hydroperoxides (PLOOHs) during ferroptosis [33]. Selenium deficiency impairs the antioxidant function of GPX4, reducing cellular resistance to ferroptosis [34]. In conjunction with reduced GSH, GPX4 reduces PLOOHs in cell membranes to harmless phospholipid alcohols (PLOHs), thereby preventing the accumulation of lipid peroxides [29, 34].

The activity of the system Xc⁻–GPX4 pathway is regulated by various factors, including intracellular redox status, iron levels, and multiple signalling pathways. For example, nuclear factor erythroid 2-related factor 2 (Nrf2) can increase the expression of GPX4 and GSH through antioxidant response elements (AREs) [35].

### Pathogen-induced ferroptosis

Pathogen-induced ferroptosis is a complex, multilayered process. Certain pathogens and their products can directly activate ferroptosis signalling pathways in host cells or disrupt intracellular iron metabolism, inducing ferroptosis. For example, *Pseudomonas aeruginosa* secretes toxic substances such as pyocyanin and pyoverdine, which promote iron accumulation and lipid peroxidation within host cells [36–38].

Some pathogens and their secreted factors promote oxidative stress and activate inflammatory responses in host cells, thereby exacerbating ferroptosis. For example, *Staphylococcus aureus* secretes α-haemolysin, which not only increases membrane permeability and facilitates iron influx but also activates the NLRP3 inflammasome, triggering the release of proinflammatory factors such as IL-1β and TNF-α, thus accelerating ferroptosis in host cells [39].

Pathogens can also induce ferroptosis by affecting host cell ferritinophagy and suppressing intracellular antioxidant systems. For example, *Salmonella enterica* secretes the SpiC protein, which inhibits ferritinophagy, preventing ferritin degradation and iron recycling, thereby increasing intracellular free iron levels and triggering ferroptosis [40]. Additionally, *Porphyromonas gingivalis* (*P. gingivalis*) secretes LPS and arginine-specific cysteine proteinases (RgpA/B), which inhibit the transcription of GPX4 [41]. Because the periodontal microbiota is composed of multiple pathogenic bacterial species, pathogen-induced cellular ferroptosis is likely important in periodontitis progression, but the mechanism remains incompletely understood.

## Ferroptosis and periodontal-related cells

The periodontium comprises the soft and hard tissues surrounding teeth and contains diverse cell types, each of which plays a vital role in maintaining periodontal health and function [42–44]. Since ferroptosis may be closely related to the progression of periodontitis, understanding the ferroptosis mechanisms of these cells within the periodontal inflammatory microenvironment could provide further insights into the pathological mechanisms of periodontitis (Table 1) [45].

**Table 1:** Ferroptosis of periodontal-related cells.

Types of periodontal cells	Functions in periodontal homoeostasis	Ferroptosis-related mechanisms in periodontitis	References
Macrophages	Clear pathogens, necrotic cells, and damaged cells to maintain homoeostasis.	Excessive uptake of aging red blood cells leads to iron accumulation in macrophages, triggering ferroptosis. Ferroptosis can result in an imbalance in the M1/M2 ratio of macrophages.	[49–60]
T cells	Participate in adaptive immunity by producing cytokines to combat infections.	Periodontitis leads to an increased proportion of CD4^+^ T cells in gingival tissue, and upon activation by *P. gingivalis*, the expression of SLC7A11 is significantly upregulated, triggering ferroptosis.	[61–65]
B cells	Produce antibodies in adaptive immunity.	The uptake of exogenous fatty acids produced by periodontal pathogens and the resulting autophagic response increase intracellular free fatty acids, promoting ferroptosis in B cells.	[66–71]
Neutrophils	The first responders in host immune defense. They can migrate from gingival tissue to the gingival crevice, protecting the host from the irritation caused by plaque biofilms.	In gingival tissue affected by periodontitis, the expression level of the ferroptosis marker SLC2A3 is significantly upregulated in infiltrating neutrophils.	[47, 72–75]
Fibroblasts	Gingival fibroblasts, in the lamina propria, maintain tissue elasticity and aid healing, while periodontal ligament fibroblasts stabilize the tooth and promote bone and cementum regeneration.	Periodontal pathogens induce ferroptosis in gingival fibroblasts, promoting inflammation and bone loss via the SLC7A11/GSH/GPX4 axis. IL-6 further drives tissue degradation, while blocking its receptor reduces bone loss. Peroxiredoxin 6 (PRDX6) mitigates inflammation and ferroptosis. High butyrate levels worsen periodontitis by promoting ferroptosis in periodontal ligament fibroblasts. Further research is needed.	[88–95]
Osteocytes	Regulate the survival and activity of osteoblasts and osteoclasts, maintaining bone homoeostasis.	In diabetic periodontitis, the high-glucose environment induces ferroptosis in osteocytes by inhibiting the SLC7A11/GPX4 axis or promoting lipid peroxidation and iron overload, leading to alveolar bone loss. Melatonin inhibits osteocyte ferroptosis by activating the Nrf2/HO-1 signalling pathway.	[96–102]
Osteoblasts	Bone formation and matrix mineralization.	Activating FtMt can alleviate ferroptosis in osteoblasts, while inhibiting FtMt promotes ferroptosis in osteoblasts through the ROS/PINK1/Parkin signalling pathway. Exosomes from vascular endothelial cells prevent osteoblast ferroptosis by inhibiting ferritinophagy.	[103–106]
Periodontal ligament stem cells	Periodontal ligament stem cells in the periodontal ligament are essential for tissue repair and regeneration due to their self-renewal and multipotent differentiation abilities.	*Fusobacterium nucleatum* induces ferroptosis in PDLSCs by increasing Fe²⁺ and proinflammatory cytokines, worsening periodontitis. lncRNA LINC00616 promotes ferroptosis via the miR-370/TFRC pathway, while downregulation of lncRNA MEG3 impedes osteogenic differentiation, further advancing the disease.	[107–111]

This table illustrates the main cell types in periodontal tissues (e.g., fibroblasts, bone tissue cells, and immune cells) and their functions; it also outlines the mechanisms of ferroptosis induced in different periodontal cells and the impact of ferroptosis on tissue inflammation and destruction. Ferroptosis, driven by iron overload, lipid peroxidation, antioxidant system dysfunction, and pathogen stimulation, affects the survival and function of these cells, ultimately exacerbating the progression of periodontitis.

### Ferroptosis and immune cells

In periodontal tissues, macrophages and neutrophils are involved in the clearance of pathogens, as are apoptotic and necrotic cells. In contrast, T cells and B cells mediate adaptive immune responses through the production of antibodies and the secretion of cytokines, thereby effectively controlling periodontal infections and inflammation [46–48].

Ferroptosis is integral to the immune system of the periodontium; it serves a dual function. First, cells undergoing ferroptosis can be recognized by immune cells, which subsequently initiate inflammatory or specific immune responses. Second, ferroptosis, which occurs within immune cells, can influence both their number and functionality to favour a proinflammatory response.

#### Macrophages

Macrophages play a crucial role in maintaining homoeostasis by phagocytosing dead or damaged cells [49]. In the periodontal inflammatory microenvironment, macrophages protect tissues by clearing necrotic cells. Toll-like receptor 2 (TLR2) on macrophages specifically recognizes oxygenated phosphatidylethanolamines on ferroptotic cell membranes, facilitating their removal [50]. This process reduces cell buildup and helps limit inflammation progression.

Macrophages are also susceptible to ferroptosis [51]. In periodontal inflammation, factors such as low haematocrit, elevated erythrocyte sedimentation rates, and the buildup of senescent red blood cells require macrophages to regulate iron homoeostasis by clearing these aged cells and recycling iron [52]. Excessive uptake of senescent red blood cells can trigger ferroptosis in macrophages, reducing their population and disrupting immune homoeostasis [53]. These findings suggest that periodontitis may exacerbate inflammation by inducing macrophage ferroptosis, although the exact mechanisms involved remain to be fully explored.

Ferroptosis can disrupt the balance between M1 and M2 macrophage polarization, aggravating inflammation. Research has shown that M1 macrophages, which are activated by *P. gingivalis*, exacerbate alveolar bone resorption by secreting proinflammatory mediators such as TNF-α [54, 55]. In contrast, M2 macrophages, which are primarily stimulated by oral commensal bacteria rather than pathogens such as *P. gingivalis* and *Aggregatibacter actinomycetemcomitans*, protect against alveolar bone loss in murine periodontitis models [56, 57]. M1 macrophages are less prone to ferroptosis and maintain proinflammatory activity because of the inhibition of arachidonate 15-lipoxygenase (ALOX15), a key enzyme in ferroptosis, by nitric oxide (NO) produced via inducible nitric oxide synthase (iNOS) [58–60]. This suppression of lipid peroxidation reduces ferroptosis in M1 macrophages.

#### T cells

T cells are essential for the adaptive immune response, with CD4^+^ T cells playing a pivotal role in periodontitis pathogenesis [61, 62]. During periodontitis progression, innate immune cells detect bacterial antigens in subgingival plaques, migrate to cervical lymph nodes, and present these antigens to activate T cells, leading to CD4^+^ T cell proliferation [61]. Unlike CD8^+^ T cells, CD4^+^ T cells are key mediators of *P. gingivalis*-induced alveolar bone loss [63]. Research has shown that naïve T cells differentiate into CD4^+^ T cells more prominently in periodontitis than in gingivitis [64]. While naïve T cells have low levels of the ferroptosis-related protein SLC7A11, its expression significantly increases upon activation by *P. gingivalis* [65]. These findings suggest that periodontal pathogens may promote periodontitis by upregulating SLC7A11 expression in CD4^+^ T cells, thereby activating the ferroptosis pathway.

#### B cells

B cells are key players in adaptive immunity, particularly in antibody production. In late-stage chronic periodontitis, B cells and plasma cells are the predominant immune cells that infiltrate affected tissues [66]. Periodontal pathogens produce substantial amounts of short-chain fatty acids (SCFAs), such as lactic acid, acetic acid, and butyric acid, which may increase B-cell susceptibility to ferroptosis [67, 68]. Research indicates that B-cell viability, especially in the B1a subtype, depends on fatty acid synthesis and storage, which are largely supported by the autophagic degradation of exogenous fatty acids [69]. In the inflammatory microenvironment, elevated short-chain fatty acids (SCFAs) may increase fatty acid levels in B cells, increasing the involvement of polyunsaturated fatty acids (PUFAs) in the Fenton reaction and promoting ferroptosis. This could intensify inflammation [70, 71]; however, the exact mechanisms by which periodontitis induces ferroptosis in B cells and drives inflammation require further investigation.

#### Neutrophils

Neutrophils are the first responders in immune defence, migrating from gingival tissues to the gingival crevice in response to chemokines and adhesion molecules, forming a protective barrier against plaque biofilms [72]. Approximately 30000 neutrophils cross periodontal tissues each minute, highlighting their essential role in immune surveillance and periodontal homoeostasis [73]. Periodontal pathogens such as *P. gingivalis* delay neutrophil apoptosis, promoting a chronic proinflammatory state that leads to tissue damage and periodontal destruction [74]. Additionally, neutrophils infiltrating periodontal tissues show elevated expression of SLC2A3, a ferroptosis marker linked to glucose transport, suggesting that neutrophils may intensify inflammation through ferroptosis [47, 75]. However, the exact mechanisms require further study.

#### Ferroptosis-related genes in periodontal immune cells

Studies utilizing the transcriptomic sequencing of gingival tissue samples from healthy individuals and patients with periodontitis have revealed the significant upregulation of several ferroptosis-related genes intimately associated with immune cell functions, such as ALOX5, XBP1, CD19, and ITGB2, within inflamed periodontal tissues [45].

5-Lipoxygenase (ALOX5), which is prominently expressed and elevated in immune cells such as macrophages and B cells within periodontal tissues, translocates from the cytoplasm to membrane structures, where it interacts with 5-lipoxygenase-activating protein (FLAP). This complex catalyzes the oxidation of PUFAs, generating lipid peroxide intermediates and subsequently inducing extensive lipid peroxidation in cellular membranes, ultimately leading to ferroptosis [76, 77]. Additionally, increased ALOX5 expression is positively correlated with cytokines that promote periodontal inflammation and alveolar bone resorption. Correspondingly, ALOX5 inhibition has been shown to attenuate inflammation, diminish immune cell infiltration, and reduce alveolar bone loss [78, 79].

X-box binding protein 1 (XBP1), a key factor in B-cell development and differentiation, is excessively activated in inflamed periodontal tissues, increasing the expression of critical B-cell chemokine genes (such as CXCL8 and CXCR4), thereby exacerbating intracellular oxidative stress and triggering ferroptosis [45, 80, 81].

The expression of CD19, a B-cell surface marker, is upregulated in inflamed gingival tissues. CD19 promotes the antigen-dependent activation of periodontal pathogens and enhances the production of the proinflammatory cytokines IL-6 and TNF-α. This cascade induces ferroptosis in B cells within periodontal tissues, accelerating tissue damage and exacerbating inflammation [82–84].

Integrin subunit beta 2 (ITGB2), which is widely expressed on macrophages, T cells, and other immune cells, contributes to increased oxidative stress when it is overexpressed. Furthermore, ITGB2 suppresses the expression of SLC7A11 (the light chain of the Xc⁻ complex), facilitating the accumulation of lipid peroxides and consequently inducing ferroptosis [85–87].

### Ferroptosis and fibroblasts

Fibroblasts primarily consist of gingival fibroblasts and periodontal ligament fibroblasts. Gingival fibroblasts are located mainly in the lamina propria of the gingiva and are responsible for synthesizing and remodelling the extracellular matrix (ECM), maintaining tissue elasticity and strength, and participating in gingival healing and repair [88, 89]. In contrast, periodontal ligament fibroblasts are situated between the tooth root and alveolar bone, where they not only contribute to the stability of the tooth but also possess the ability to differentiate into osteoblasts and cementoblasts, thereby promoting the regeneration and remodelling of bone and cementum [90, 91].

Periodontal pathogens promote periodontal inflammation by inducing ferroptosis in gingival fibroblasts. In vivo and in vitro studies have shown that *P. gingivalis* triggers ferroptosis in gingival fibroblasts by inhibiting the SLC7A11/GSH/GPX4 axis. The increase in IL-6 resulting from ferroptosis in gingival fibroblasts further promotes inflammation and bone loss in periodontal tissues [92]. IL-6 signalling upregulates the expression of MMP-1, monocyte chemoattractant protein-1 (MCP-1), and interleukin-8 (IL-8), which together drive collagen degradation and inflammatory cell infiltration [12, 93]. Blocking the IL-6 receptor has been shown to reduce inflammatory bone loss in periodontitis.

Peroxiredoxin 6 (PRDX6), which is regulated by Nrf2, helps reduce LPS-induced inflammation and ferroptosis, thereby slowing periodontitis progression [94]. In deep periodontal pockets, high butyrate levels promote ferroptosis in periodontal ligament fibroblasts via nuclear receptor coactivator 4 (NCOA4), worsening periodontitis [95]. However, research on ferroptosis in these fibroblasts and its link to periodontal inflammation remains limited, underscoring the need for further investigation.

### Ferroptosis and bone tissue cells

Osteocytes, osteoblasts, and osteoclasts are present within the alveolar bone, and they collectively regulate the remodelling of alveolar bone through the synthesis, mineralization, and resorption of the bone matrix. Among these, osteocytes account for approximately 95% of the cells in the alveolar bone and play a crucial role in bone homoeostasis by regulating the activity of osteoblasts and osteoclasts [96–98].

In periodontitis, however, osteocytes disrupt this balance by secreting proinflammatory cytokines and undergoing apoptosis, which leads to increased osteoclast activity, reduced osteoblast differentiation, and impaired extracellular matrix mineralization [99, 100]. In diabetic periodontitis, ferroptosis in osteocytes—mediated by the suppression of the SLC7A11/GPX4 pathway—contributes to alveolar bone deterioration [101]. Hyperglycaemia exacerbates this process by promoting lipid peroxidation and iron accumulation [102]. Additionally, advanced glycation end products (AGEs) induce ferroptosis in osteoblasts, further intensifying bone loss [103]. Osteoblast death releases inflammatory mediators such as sclerostin (SOST), RANKL, IL-1β, and TNF-α, which impair osteoblast function and promote osteoclast formation, accelerating bone loss [104].

Mitochondrial ferritin (FtMt) plays a key role in regulating ferroptosis in osteoblasts. FtMt activation alleviates ferroptosis, while its inhibition induces mitophagy via the ROS/PINK1/Parkin pathway, thereby promoting ferroptosis. FtMt is essential for bone remodelling and preventing bone loss; however, its role in periodontal inflammation remains unexplored [105].

Therapeutically, melatonin has been shown to inhibit osteoblast ferroptosis and promote osteogenesis through the Nrf2/HO-1 pathway [102]. Additionally, exosomes from vascular endothelial cells prevent osteoblast ferroptosis by inhibiting ferritinophagy, suggesting promising strategies to mitigate bone loss and improve bone health in periodontitis [106].

### Ferroptosis and periodontal ligament stem cells

Periodontal ligament stem cells (PDLSCs) are present in the periodontal ligament and possess self-renewal and multipotent differentiation capabilities. These cells are crucial for the repair and regeneration of periodontal tissues [107–109].

Recent research indicates that *Fusobacterium nucleatum* not only impedes the proliferation of PDLSCs but also triggers ferroptosis by increasing the intracellular free Fe²⁺ concentration. This ferroptosis induction leads to an increase in the levels of proinflammatory cytokines, including IL-1β, IL-6, and IL-8, thereby exacerbating the progression of periodontitis [110].

Both in vitro and in vivo investigations revealed that the long noncoding RNA (lncRNA) LINC00616 facilitates ferroptosis in PDLSCs via the miR-370/TFRC signalling pathway. In the context of periodontitis, the downregulation of lncRNA MEG3 expression impedes the osteogenic differentiation of PDLSCs through the miR-27a-3p/IGF1 axis, further contributing to disease progression [111].

## Mechanisms by which ferroptosis promotes the development of periodontitis

**Fig. 2:**
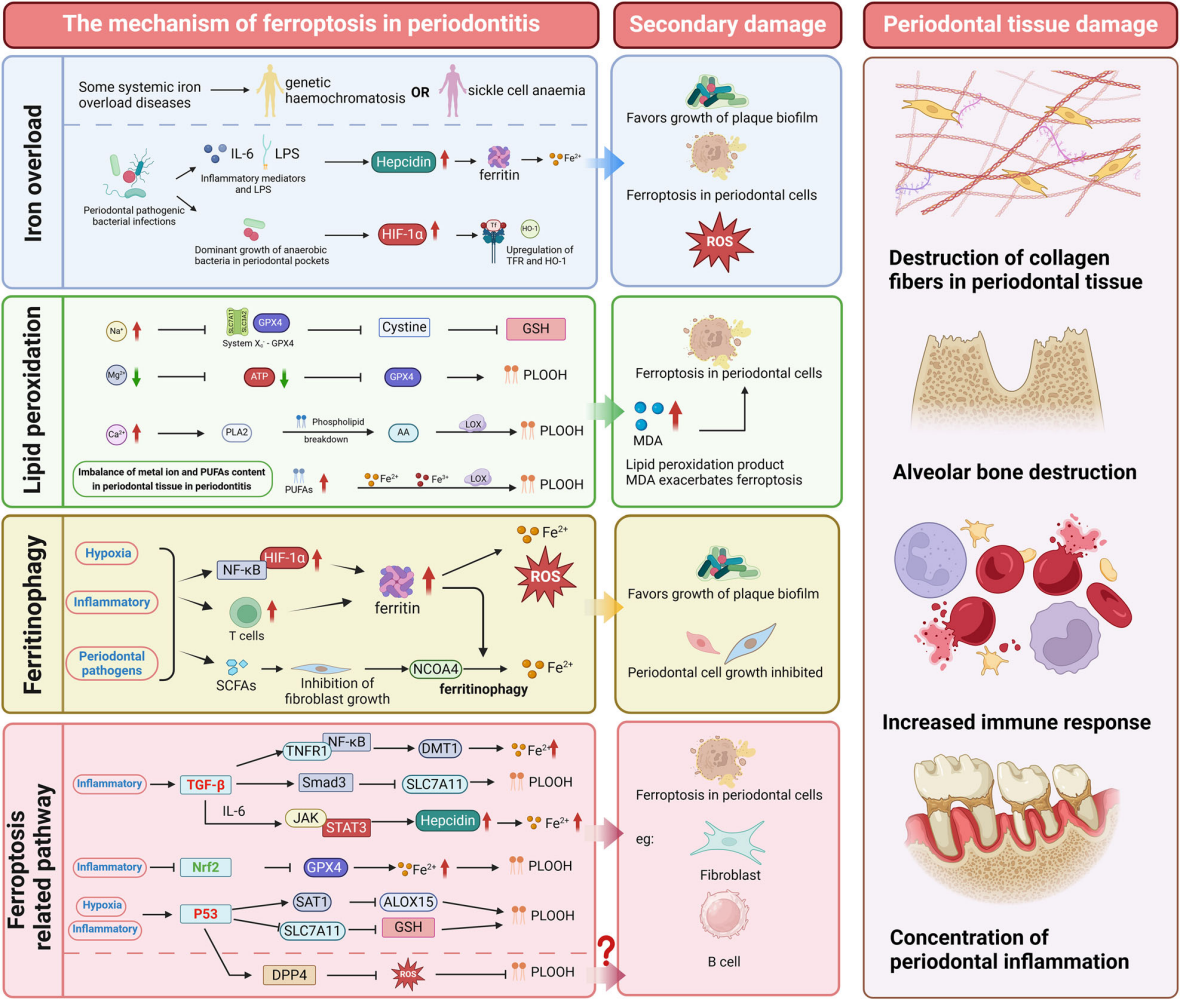
Mechanisms of ferroptosis in periodontitis progression. In periodontal tissue cells, the mechanisms driving ferroptosis primarily include iron overload, lipid peroxidation, increased ferritinophagy, and the activation of ferroptosis-related pathways. Certain systemic diseases and the inflammatory microenvironment in periodontitis contribute to localized iron overload, which induces ferroptosis in periodontal cells. During periodontitis, changes in the metal ion content in saliva and gingival crevicular fluid, along with increased PUFA levels, increase lipid peroxidation in periodontal cells. Under inflammatory, pathogenic, and hypoxic conditions, ferritinophagy within periodontal cells increases, leading to abnormal intracellular iron accumulation and subsequent ferroptosis. Moreover, multiple ferroptosis-related pathways are activated in the periodontal inflammatory microenvironment, while ferroptosis defence pathways (e.g., the Nrf2 pathway) are suppressed. These mechanisms render periodontal cells more prone to ferroptosis, further damaging periodontal tissues and exacerbating periodontitis.

Numerous studies have demonstrated that ferroptosis is associated with various inflammatory diseases. Periodontitis, a chronic inflammatory disease initiated by plaque microorganisms, leads to the progressive destruction of periodontal supporting tissues. However, research on the role of ferroptosis in the pathogenesis of periodontitis remains limited (Fig. 2).

### Iron overload driven by a high-iron environment and periodontal pathogens

Elevated iron concentrations within periodontal tissues can accelerate the progression of periodontal inflammation [10, 112]. Certain systemic iron overload diseases, such as genetic haemochromatosis [113] and sickle cell anaemia [114], significantly increase the severity of periodontitis.

Within 12 h of periodontal infection, local free iron concentrations increase significantly, facilitating biofilm maturation and growth [115]. For example, *P. gingivalis* and *Prevotella intermedia* exploit host iron-binding proteins to access iron. By degrading these proteins, they not only secure the iron needed for their growth but also release iron fragments and free iron, leading to localized iron overload [41, 116].

Additionally, the increase in anaerobic bacteria within periodontal pockets causes local hypoxia, leading to the high expression of hypoxia-inducible factor-1α (HIF-1α) in inflamed periodontal tissues [117]. HIF-1α can upregulate the expression of transferrin receptor (TFR) and HO-1, further increasing intracellular iron levels and inducing ferroptosis in periodontal cells [118].

### Enhanced lipid peroxidation and antioxidant system impairment

Research has shown that lipid peroxide levels are significantly increased in the saliva and gingival crevicular fluid of chronic periodontitis patients [119]. Additionally, trace metal ions such as Fe²⁺, Na⁺, Mg²⁺, and Ca²⁺ in saliva undergo notable changes in these patients, with these ions playing crucial roles in regulating redox reactions and lipid peroxidation in periodontitis [120].

In the periodontal microenvironment, increased Fe²⁺ directly induces ferroptosis in periodontal cells, promoting inflammation progression [114, 121]. Additionally, elevated Na⁺ concentrations may reduce the activity of the system Xc^−^–GPX4 antioxidant pathway, decreasing cystine uptake, reducing GSH synthesis, and weakening cellular antioxidant defences [122]. A reduction in Mg²⁺ levels may lead to ATP deficiency in macrophages within periodontal tissues, subsequently decreasing GPX4 and GSH synthesis and diminishing the cellular antioxidant capacity [123]. Elevated Ca²⁺ levels activate phospholipase A2 (PLA2), promoting the breakdown of phospholipids and generating arachidonic acid (AA). Under the action of LOX, AA is converted into lipid peroxides, thereby increasing lipid peroxidation [124].

Increased intracellular ROS production further exacerbates lipid peroxidation. In the periodontal microenvironment, neutrophils produce substantial amounts of ROS via NADPH oxidase (NOX)-catalyzed respiratory bursts, accelerating lipid peroxidation and ferroptosis [118].

Malondialdehyde (MDA), a byproduct of lipid peroxidation and a ferroptosis marker, is significantly elevated in inflamed periodontal tissues. Upon stimulation by *P. gingivalis* lipopolysaccharides, ferroptosis markers (ROS, Fe²⁺, and MDA) increase markedly in periodontal ligament stem cells, whereas GSH levels in the antioxidant system significantly decrease [125]. Treatment with the ferroptosis inhibitor ferrostatin-1 suppresses ferroptosis in these cells, significantly reducing MDA expression [126].

The lipid peroxidation mechanism of ferroptosis suggests that PUFAs undergo peroxidation under the influence of iron (both Fe³⁺ and Fe²⁺) and LOX, generating products, including MDA, which then induce ferroptosis [127]. Studies have shown that periodontal inflammation can alter the serum levels of n-3 and n-6 PUFAs. However, there is currently no direct evidence that *P. gingivalis* or its metabolites directly impact MDA levels via the PUFA/LOX pathway, warranting further investigation [128].

### Increased ferritinophagy

Studies indicate that the sources of ferritin in the periodontal inflammatory microenvironment may be mainly divided into two aspects. First, hypoxia-inducible factors (HIFs), regulated by NF-κB in inflamed periodontal tissues, are key regulators of iron homoeostasis-related proteins, including ferritin, TFR1, HO-1, divalent metal transporter 1 (DMT1), and erythropoietin [129]. On the other hand, inflamed periodontal tissues are infiltrated by T cells, which possess a strong capacity to synthesize and secrete ferritin. However, direct evidence confirming that T cells are the source of ferritin in inflamed periodontal tissues has not yet been reported [130].

Ferritinophagy is an autophagic process that releases iron by degrading ferritin. Periodontal pathogens may increase ferritinophagy, increasing Fe²⁺ levels in human periodontal ligament fibroblasts [131]. In periodontitis, oral microbiome dysbiosis occurs, with increased abundances of anaerobes such as *P. gingivalis*, *Actinomyces*, and *Fusobacterium nucleatum*, resulting in the production of high levels of short-chain fatty acids (SCFAs), such as lactate, acetate, and butyrate, which are correlated with periodontitis severity [67]. Butyrate, a key SCFA in periodontal pockets, can inhibit fibroblast growth, promote NCOA4 expression, induce ferritin autophagy, release free iron, deplete GSH and GPX4, increase ACSL4 expression, and trigger lipid peroxidation [132]. Concurrently, periodontal pathogens utilize ferritin breakdown for metabolic and reproductive needs, supporting their growth and virulence [133].

### Activation of ferroptosis-related signalling pathways

Ferroptosis in periodontal cells is regulated by multiple signalling pathways, including key pathways such as the TGF-β, Nrf2, and P53 pathways.

#### TGF-β pathway

Periodontal pathogens in the inflammatory microenvironment can stimulate periodontal cells such as fibroblasts and B cells to highly express transforming growth factor-β (TGF-β), which, via the TNFR1/NF-κB signalling pathway, exacerbates oxidative stress and promotes the expression of iron metabolism-related proteins (e.g., DMT1), leading to excessive intracellular Fe²⁺ accumulation [134]. TGF-β1 can induce ferroptosis by activating the Smad3 pathway to inhibit SLC7A11 expression [135]. Additionally, TGF-β synergizes with IL-6, activating the JAK/STAT3 signalling pathway to increase the expression of intracellular hepcidin, further increasing Fe²⁺ accumulation [136]. TGF-β also regulates the polarization state of macrophages in the periodontal inflammatory microenvironment, particularly by promoting M2 polarization, causing these macrophages to express high levels of iron uptake proteins (e.g., CD163), thereby increasing iron uptake and storage, increasing intracellular Fe²⁺ concentrations, and further driving ferroptosis [137].

#### Nrf2 pathway

Nrf2 is a key transcription factor in cellular responses to oxidative stress and plays a crucial role in regulating antioxidant responses and maintaining the intracellular redox balance [138]. In periodontitis, the Nrf2 signalling pathway is suppressed, leading to reduced antioxidant capacity. By regulating various antioxidant and iron metabolism-related genes, such as GPX4 and ferritin, Nrf2 protects cells from oxidative damage and iron overload [125]. Studies have shown that activating the Nrf2 pathway can increase GPX4 expression, reduce lipid peroxide accumulation, and inhibit ferroptosis [139]. Additionally, Nrf2 enhances the cellular antioxidant capacity by upregulating the expression of antioxidant enzymes and reducing ROS production, thereby alleviating periodontal inflammation and protecting periodontal tissues from further damage [140].

#### p53 pathway

p53 is a tumour suppressor protein that plays a central role in cellular responses to stressors such as DNA damage and hypoxia. In the hypoxic and inflammatory environment of periodontitis, p53 expression is induced, and p53 levels are positively correlated with periodontitis severity [141]. Research shows that p53 plays dual roles in both promoting and inhibiting ferroptosis: on the one hand, it promotes ferroptosis by downregulating the expression of SLC7A11, a negative ferroptosis regulator, and by enhancing the expression of spermidine/spermine N1-acetyltransferase 1 (SAT1), which upregulates ALOX15 expression to drive lipid peroxidation [142].

Conversely, p53 can inhibit ferroptosis by modulating dipeptidyl peptidase-4 (DPP4) activity to reduce oxidative stress and by inducing CDKN1A/p21 expression [143]. Despite these findings, the molecular mechanisms of p53 in periodontitis-related ferroptosis remain underexplored and warrant further study.

## Periodontitis contributes to the progression of systemic diseases by inducing ferroptosis in distant organs

**Fig. 3:**
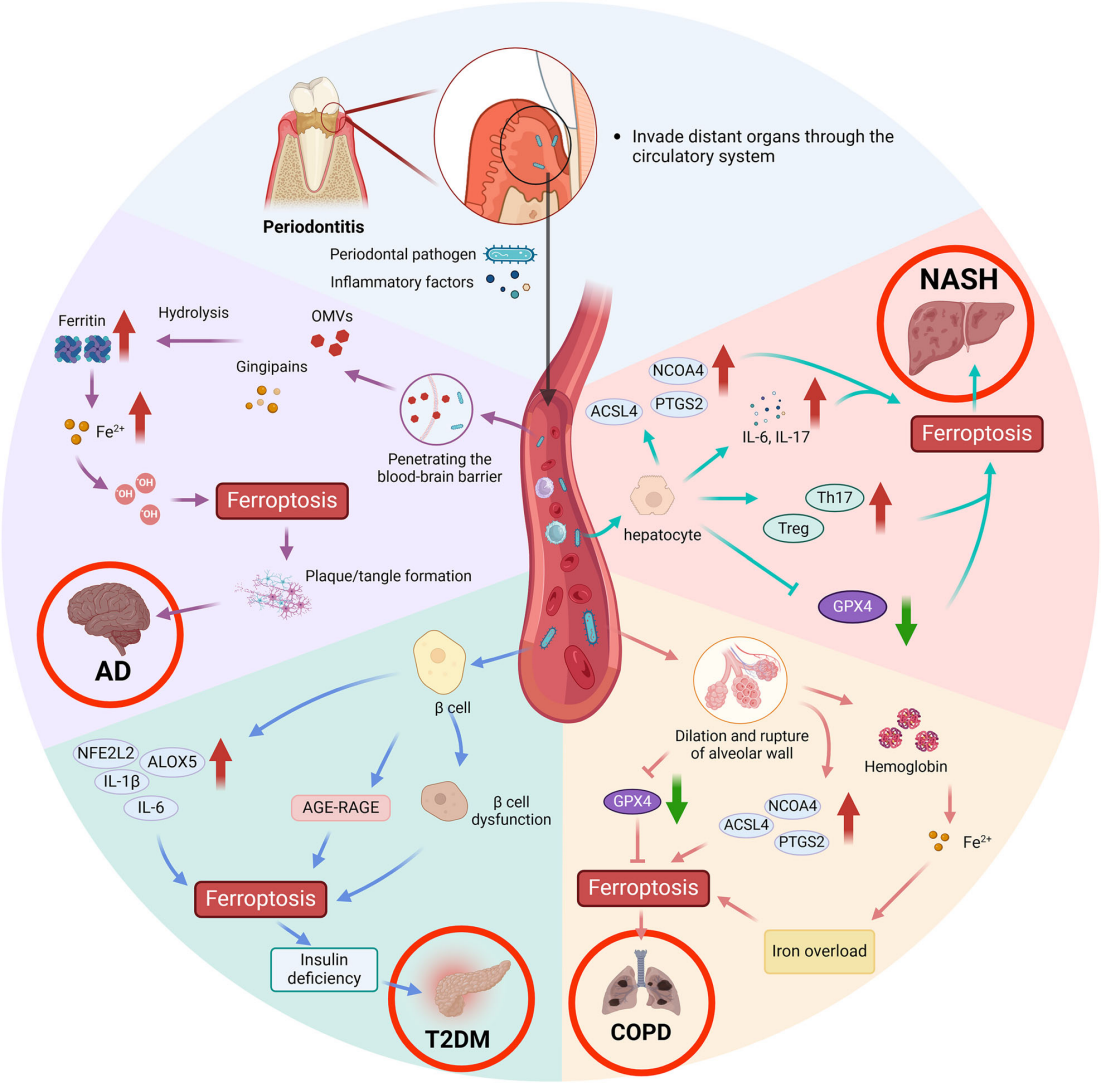
Potential Pathways by which Periodontitis Influences Systemic Diseases through Ferroptosis Mechanisms. Periodontal pathogens and their products can spread through the bloodstream to distant organs, inducing ferroptosis in those cells. *P. gingivalis* can cross the blood‒brain barrier to reach brain tissue and secrete LPS and OMVs, inducing ferroptosis in neurons and promoting the progression of Alzheimer’s disease (AD); it can damage pancreatic β cells by upregulating the expression of oxidative enzymes such as NFE2L2 and ALOX5, facilitating intracellular lipid peroxidation. Additionally, *P. gingivalis* can increase the degradation of haem in alveolar cells, leading to elevated intracellular iron levels and subsequent ferroptosis; furthermore, it can upregulate the expression of key enzymes involved in lipid peroxidation, such as ACSL4, in hepatocytes, promoting ferroptosis. Thus, ferroptosis may represent a mechanism by which periodontitis contributes to the development of systemic diseases.

Periodontitis, a major risk factor for systemic diseases, is associated with an increased incidence of Alzheimer’s disease, nonalcoholic steatohepatitis, chronic obstructive pulmonary disease, and diabetes. Studies indicate that periodontal pathogens and their metabolites can enter systemic circulation through ulcerated periodontal tissues, inducing ferroptosis in distant organs and promoting systemic disease progression. However, research on the role of periodontitis in regulating systemic diseases via ferroptosis remains limited, highlighting the need for further investigation (Fig. 3).

### Periodontitis promotes AD via ferroptosis

Chronic periodontitis is considered a risk factor for Alzheimer’s disease (AD). In the brain tissue of AD patients, *P. gingivalis* and its products, such as lipopolysaccharide (LPS), gingipains, and outer membrane vesicles (OMVs), have been found. This bacterium can enter the circulatory system through periodontal tissue and cross the blood‒brain barrier to reach the brain [144].

*P. gingivalis* has the ability to take up iron and can release iron by degrading haem, increasing the levels of free iron in tissues [144]. When this bacterium is phagocytosed by immune cells in the brain (such as macrophages), it releases large amounts of iron ions and ferritin, leading to a sharp increase in the intracellular iron level and resulting in ferroptosis [145].

Moreover, after OMVs enter brain tissue, the gingipains they carry can hydrolyze transferrin in neuronal cells, releasing Fe³⁺ and Fe²⁺, which causes intracellular iron overload [146, 147]. LPS from *P. gingivalis* can induce the expression of NLRP3 in neuronal cells, and in vitro studies have shown that NLRP3 agonists can promote ferroptosis in mouse neuronal cells [148].

### Periodontitis promotes NASH via ferroptosis

Research has shown that the relationship between periodontitis and nonalcoholic steatohepatitis (NASH) is not merely coincidental but involves mutual influence through multiple mechanisms.

In vivo experiments indicate that mice orally administered *P. gingivalis* are more susceptible to developing NASH, with elevated iron levels in the liver, significantly increased mRNA and protein expression of the ferroptosis markers NCOA4 and PTGS2, and decreased mRNA and protein expression of GPX4 [149]. Additionally, an imbalance in the Th17/Treg ratio was observed in the liver and spleen. Mice treated with the ferroptosis inhibitor Fer-1 presented a markedly lower incidence of NASH, with ferroptosis in liver tissue significantly suppressed [150]. Subsequent studies revealed that *P. gingivalis* can activate the NF-κB signalling pathway in hepatic L-02 cells in vitro, leading to ferroptosis [151]. These findings suggest that *P. gingivalis* may promote NASH progression by inducing ferroptosis in hepatocytes.

### Periodontitis promotes COPD via ferroptosis

*P. gingivalis* in periodontitis can promote the progression of chronic obstructive pulmonary disease (COPD) through multiple pathways, including the induction of ferroptosis in lung tissue cells [152, 153].

Recent studies have shown that, in addition to traditional factors such as long-term smoking and air pollution, chronic inflammatory diseases such as periodontitis can promote the progression of COPD [154, 155]. In vivo experiments demonstrated that mice in a combined periodontitis and COPD group exhibited significant alveolar wall expansion, rupture, and pulmonary iron accumulation [154]. Additionally, the expression of the ferroptosis-related genes ACSL4, PTGS2, and NCOA4 is significantly increased, whereas GPX4 mRNA expression is markedly reduced [156]. Further analysis revealed that, in patients with periodontitis, lung tissue is overloaded with iron, resulting in increased ROS generation [156]. The inflammatory response of pulmonary macrophages may induce ferroptosis in epithelial cells, thereby worsening COPD severity.

### Periodontitis promotes T2DM via ferroptosis

Periodontitis is recognized as the sixth most common complication of type 2 diabetes (T2DM). Ferroptosis induced by periodontitis can exacerbate insulin resistance and increase the risk of complications in T2DM patients through immune-inflammatory responses and the AGE-RAGE signalling pathway [14, 157]. Compared with T2DM patients with healthy periodontal conditions, T2DM patients with periodontitis exhibit significantly higher expression of ferroptosis-related genes, such as IL-1β, IL-6, NFE2L2, and ALOX5, in pancreatic tissue [158]. Additionally, in vitro experiments revealed that *P. gingivalis* and its metabolites can impair human pancreatic β cell function [158]. Thus, ferroptosis induction may be one mechanism by which periodontitis increases the severity of T2DM.

## Discussion and outlook

Although ferroptosis is one of the mechanisms promoting the progression of periodontitis, the upstream inducing factors remain incompletely understood. Among the known triggers, periodontal pathogens, particularly *P. gingivalis*, are the most commonly cited. However, the specific components (such as LPS, OMVs, etc.) and molecular pathways through which these pathogens induce ferroptosis in periodontal tissue cells have yet to be fully elucidated. Future research could focus more on investigating pathogen virulence factors or metabolic products that regulate ferroptosis in periodontal tissue cells.

Additionally, the role of ferroptosis in periodontitis may vary between individuals. Differences in genetic background, immune status, and microbiota composition among patients could result in heterogeneous manifestations of ferroptosis in periodontal tissues. For example, some patients may be more susceptible to iron-dependent lipid peroxidation due to lower expression levels of key genes such as SLC7A11 or GPX4, potentially exacerbating tissue destruction in periodontal tissues. Moreover, systemic diseases such as diabetes and hypertension may increase oxidative stress and iron ion levels in periodontal tissues, promoting ferroptosis. Future studies should consider individual differences in susceptibility to ferroptosis and explore ferroptosis regulatory networks related to patient genetic backgrounds and metabolic states, providing a basis for the development of personalized intervention strategies.

Third, while therapeutic strategies targeting ferroptosis, such as antioxidants, iron chelators, and gene therapy, have shown preliminary promise, their clinical translation requires caution. For example, lipid peroxidation inhibitors may suppress ferroptosis in periodontal tissue cells but could also disrupt physiological oxidative signalling between periodontal cells, potentially delaying wound healing. Therefore, a balance must be reached between inhibiting pathogenic ferroptosis and maintaining essential cellular redox signalling.

Further in vivo and clinical studies are needed to assess the safety, efficacy, and impact of ferroptosis interventions for oral diseases and even systemic conditions.
